# Indicators for Intellectual Disability Where No Formal Diagnosis Exists but Nursing Knowledge Demonstrates Grounds for a Formal Assessment: A Scoping Review

**DOI:** 10.3390/healthcare13131489

**Published:** 2025-06-21

**Authors:** Owen Doody, Kumaresan Cithambaram, Judy Ryan, Ruth Ryan, Martina Conway, Deirdre Corby

**Affiliations:** 1 School of Nursing and Midwifery, University of Limerick, V94 T9PX Limerick, Ireland; ruth.ryan@ul.ie; 2 Department of Nursing and Healthcare, Technological University of the Shannon, N37 HD68 Athlone, Ireland; kumaresan.cithambaram@tus.ie; 3 Nursing and Midwifery Planning and Development, Health Service Executive, R95 DK07 Kilkenny, Ireland; judy.ryan@hse.ie; 4 School of Nursing, Psychotherapy and Community Health, Dublin City University, D09 Y8VX Dublin, Ireland; martina.conway@dcu.ie (M.C.); deirdre.corby@dcu.ie (D.C.)

**Keywords:** undiagnosed, intellectual disability, literature review

## Abstract

Globally, 1–3% of the population has an intellectual disability, but some remain undiagnosed, resulting in limited access to essential health and social care services, poor health outcomes, and higher risks of homelessness, substance abuse, and imprisonment. A formal diagnosis enables early intervention and support. A scoping review was conducted to explore research on undiagnosed intellectual disability, screening processes, and identifying indicators. Method: The scoping review search was conducted using academic databases such as CINAHL, PsycINFO, Scopus, and PubMed, along with eight grey literature sources. In addition, the reference lists of the included studies were explored. Information specialists supported and guided the search process. The search included qualitative, quantitative, review, and mixed-method research studies published in English between 2000 and 2024. Two reviewers screened papers for eligibility by title, abstract, and full text. Result: A total of 11,475 papers were screened, with 57 papers from various countries included in the review. Indicators of intellectual disability were identified in three settings: (1) educational settings (preschool, primary, and secondary schools); (2) social care services, including homeless and community services; and (3) criminal services, such as courts, probation, and prisons, highlighting the wide applicability of findings. These indicators were linked to “experiences”, “behaviours”, “challenges”, and “observations”. Conclusion: This review emphasised the importance of early diagnosis by adopting appropriate assessment tools, which require national screening policies. It also highlighted the essential role of professionals working in intellectual disability services in identifying and supporting undiagnosed individuals, particularly within high-risk populations. Implication: The review’s findings will guide policy, practice, and research recommendations for enhancing the early identification of people with intellectual disabilities.

## 1. Introduction

Intellectual disability presents a significant impairment in general intellectual and adaptive functioning originating in childhood [1]. The global intellectual disability prevalence rates are accepted as being between 1% and 3% of the population, and there is a segment of the population who show indicators of intellectual disability but have no formal diagnosis [2]. The absence of a diagnosis prevents access to appropriate services, necessary care adjustments, and specialised expertise, increasing the risk of involvement with the criminal justice system or becoming homeless. Research indicates that individuals with intellectual disabilities are disproportionately represented in homelessness [3] and criminal justice [4] figures. Those with undiagnosed intellectual disability are vulnerable to these issues due to their limited societal interactions [5], and they suffer poorer health outcomes [6] as a result. The absence of a formal diagnosis significantly impedes access to services, and national data in Ireland, for example, identifies 28,998 people as having an intellectual disability on the National Ability Supports System database [7]. On the other hand, the Irish census data (CSO) identifies 109,288 people with intellectual disability [8]. This highlights a difference of 80,290 individuals and may be because the National Ability Supports System is only a register of those known to provider services or receiving recognised support, while the CSO figures include self-reported data, and this figure may capture a wider range of individuals not formally diagnosed or registered.

Emerging research suggests that undiagnosed intellectual disability is not rare, especially among adults within mental health and criminal justice settings; a significant proportion of individuals in these systems meet the criteria for intellectual disability but lack a formal diagnosis [9,10]. Further, a substantial number of undiagnosed mild and borderline people with intellectual disability are at risk of adverse life outcomes, including poor physical and mental health, poverty, insecure employment, and involvement with the justice system. These risks are largely attributed to the under-recognition of their cognitive impairments in the design of care and support services [11,12]. Epidemiological studies further suggest that the prevalence of intellectual disability among adults is likely underestimated, particularly among socioeconomically disadvantaged and minority groups [13]. These findings underscore the need for improved screening pathways and further epidemiological work to determine the prevalence and characteristics of undiagnosed populations. Undiagnosed intellectual disability presents challenges such as limited access to services, exacerbated mental health issues, homelessness, offending behaviour, addiction, and social stigma [14,15]. Timely diagnosis is crucial for accessing support services, requiring a comprehensive approach [3,16] and indicators for identifying undiagnosed intellectual disability including developmental milestones, behavioural patterns, communication skills, and academic performance [17]. Given the knowledge that undiagnosed and borderline intellectual disability figures are not captured, screening of individuals upon entry into health and social care services should occur where indicated [14,18]. Addressing undiagnosed intellectual disability is a compassionate and strategic response that aligns with global goals for inclusivity and equal opportunities [4]. The reasons for underdiagnosis can result not only from limited access to services but also the subtle presentation of mild intellectual disability, lack of awareness among general healthcare providers, and the influence of social stigma, which may prevent individuals or their families from seeking evaluation. While some studies have explored specific aspects of undiagnosed intellectual disability, this review uniquely maps indicators and screening tools across diverse sectors of education, social care, and criminal justice, offering a comprehensive foundation for practice and policy development. Thus, a scoping review is warranted to systematically explore the extent and nature of the existing research on undiagnosed intellectual disability, the screening process, and to identify indicators of intellectual disability. While previous studies have examined aspects of intellectual disability, a comprehensive mapping of indicators for undiagnosed cases across diverse settings has been lacking. This paper presents such a review, which is the first in this area to the authors’ knowledge, and the results will inform policy, practice, and research recommendations to improve outcomes for this vulnerable population group.

## 2. Materials and Methods

A scoping review was chosen to provide a comprehensive overview of the issues of undiagnosed intellectual disability and to map the existing literature pertinent to the research questions without being limited by study quality or design. Scoping reviews organise publications by categorising various elements of the literature such as study design, population, setting, intervention, theoretical or conceptual framework, key aspects, and outcomes. This approach helps to understand the breadth and depth of the literature. For this scoping review, the methodological framework proposed by Arksey and O’Malley [19] was employed, involving a six-step process: (1) identifying the research question; (2) identifying relevant studies; (3) selecting studies; (4) charting the data; (5) collating, summarising, and reporting the results; and (6) consulting with stakeholders/PPI involvement. The findings are presented through a narrative and tables [20]. This scoping review protocol was registered with the Open Science Framework (OSF) (Registration DOI: 10.17605/OSF.IO/SM495) on [8 April 2024]. The registration includes detailed information about the review objectives, eligibility criteria, search strategy, and data extraction procedures. The protocol is publicly accessible at https://doi.org/10.17605/OSF.IO/SM495.

### 2.1. Identifying the Research Question

To meet the aim of this review, the authors identified the following questions: (1) what indicators currently exist for intellectual disability when no formal diagnosis is present, (2) what screening tools are currently in use to screen for intellectual disability when no formal diagnosis is present, (3) who administers these tools, and (4) are there particular marginalised groups identified as being at risk.

### 2.2. Identifying Relevant Studies

A wide range of search terms was used to identify the breadth of the literature. This process included the use of open search terms and Boolean operators. Searches were completed using thesaurus terms search, title or abstract searches, and the subsequent combination of search strings (Table 1). Four electronic databases were searched (CINAHL, PsycINFO, Scopus, PubMed) along with eight grey literature sources (DANS, Grey Literature Report, NICE, CADTH, WHO Global Index Medicus, Clinicaltrials.gov, WHO ICTRP, Trip database). In addition, the reference lists (backward citation chaining) and citations (forward citation chaining) of studies included in the review were explored. Information specialists (two librarians) supported and guided the search process. The timeframe of 2000 to 2024 was chosen to capture developments in intellectual disability screening aligned with evolving service models, policies on inclusion, contemporary diagnostic practices, modern definitions of intellectual disability, and increased emphasis on inclusion and early intervention from the early 2000s onward. The search strategy was designed to ensure both breadth and specificity by combining three core concept clusters: (S1) terms for intellectual disability, (S2) screening or identification methods, and (S3) risk settings and associated factors. These clusters were joined using Boolean operators (S1 AND S2 AND S3) to focus the search on the literature at the intersection of these areas.

### 2.3. Selecting Studies

**Figure 1:**
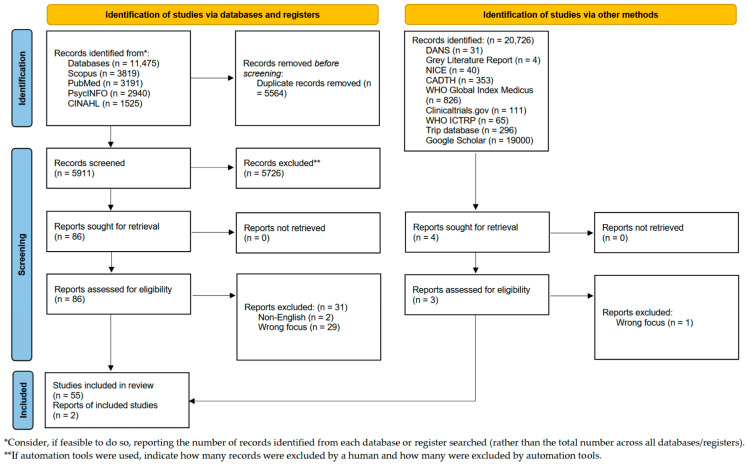
PRISMA 2020 flow diagram. Source: [21].

Database searching yielded 11,475 results, which were uploaded to Covidence, where duplicates were removed (*n* = 5564), and screening was conducted against the inclusion and exclusion criteria (Table 2). The remaining 5911 were screened at the title and abstract stage, resulting in 5825 being removed and the remaining 86 going forward to the full-text review. The grey literature search process identified 1726 results, and after initial screening (title and abstract), only 2 went forward for full-text review. Paired reviewers conducted the screening process; any disagreements between paired reviewers during title/abstract or full-text screening were resolved through discussion. If consensus could not be reached, a third reviewer was consulted to adjudicate. The search process is shown in a Preferred Reporting Items for Systematic Reviews and Meta-Analyses (PRISMA) flow diagram [21] in Figure 1, and the review was reported in line with the PRISMA extension for Scoping Reviews Checklist [22]. A full-text review resulted in 31 papers being excluded and 57 being included in this review. Common reasons for full-text exclusions included a focus on already diagnosed individuals, studies assessing treatment outcomes rather than screening, or a lack of indicators.

### 2.4. Charting the Data

A narrative outline and tables are used to present the data from this review. Data were charted into a data extraction table (Table 3) addressing the key questions identified to meet the aim of the review along with author, year, title, country, aim of study, and methodology. To assist in plotting the data, a content analysis process was conducted by paired reviewers following the steps of Colorafi and Evans [23]: (1) create a coding framework, (2) add codes and memos, (3) apply the first level of coding, (4) categorise codes and apply the second level of coding, (5) revise and redefine codes, (6) add memos, (7) visualise data, and (8) represent the data.

### 2.5. Collating, Summarising, and Reporting the Results

The findings of this review were charted, collated, summarised, and reported based on the researchers’ analysis of the included studies. This flexible approach allowed for ordering, coding, categorising, and summarising data to be presented under the key questions identified to meet the aim of the review and make recommendations to inform policy, practice, and research.

### 2.6. Consulting with Stakeholders

Integrating expert consultation within the scoping review process is emphasised, but this step is often overlooked [24]. An oversight group provided valuable insight into their experiences and perspectives, and members represented those in senior nursing positions as well as policymakers and influencers within the HSE and the Irish Prison Service. In addition, two PPI members provided consultation into the process of searching (terms), interpretation (results), presentation (findings), and recommendations (policy/practice/research) for this scoping review. One PPI member works with socially marginalised populations exhibiting indicators of intellectual disability, and the second PPI member is an expert by experience and a disability advocate.

**Table 3:** Data extraction table.

Author, Year, Country	Methodology	Aim/Objectives	Key Findings	Indicators Identified
[25] Ali and Galloway (2016) UK	Literature review.	To outline and report on the initial development of a screening tool for offenders with suspected intellectual disability (ID) known as the rapid assessment of potential intellectual disability (RAPID).	All indicated the tool to be an effective way of identifying ID and vulnerability and allowed professionals to think about mental health and ID pathology in ways that they had not previously. The authors recognised that the inclusion of a short training package about its use in addition to general ID awareness would be advantageous in promoting good practice and consistency.	Difficulty with academic work at school. Previously had a classroom assistant in school. Attended a special needs school. Attended a special needs class. Received extra help with reading, writing, and doing maths. Help now in form filling. Help with household tasks, managing money, using public transport. Previously identified or diagnosed with ID. Previously identified with learning difficulties, dyslexia, ADHD, or autism.
[26] Alramamneh and Al-Sabayleh (2024) Jordan	Cross-sectional study.	To explore and articulate the indicators of learning disabilities among kindergarden students in Amman as identified and experienced by teachers.	Of the teachers, 70% agreed with “difficulty following instructions”, 71% agreed with poor academic performance, 68.3% agreed with behavioural issues, and 59.6% with social interaction problems.	Difficulty following instructions. Poor academic performance. Behavioural issues and social interaction problems.
[27] Bhandari et al. (2015) Australia	Randomized controlled trial.	To describe social circumstances and patterns of substance use and substance-related harm in soon-to-be-released prisoners with ID compared with those without ID to understand the health-related needs of the ID group compared with the mainstream prison population.	A total of 316 participants (24%) scored below 85. Overall, 115 participants (9%) were identified as having an ID; of these participants, 108 (93%) reported attending a special school and 56 (49%) reported having been diagnosed with ID.	Having attended a special school. Having received a diagnosis of ID from a healthcare professional.
[28] Billstedt et al. (2017) Sweden	Prevalence study.	To map prevalence and clinical characteristics of neurodevelopment disorders (NDDs) among young adult male violent offenders in prison and compare young violent offenders with and without NDDs regarding childhood adversities, school age adjustment, and criminal history.	Out of 265 participants, 55 (22%, missing data in 4) had a General Ability Index score between 71 and 84, indicating borderline intellectual functioning, and 3 participants received a score at or below IQ 70 and a Global Assessment of Functioning (GAF) score < 70, indicating ID.	Difficulties in planning future tasks, judging appropriate social behaviour, learning a new skill.
[29] Board et al. (2015) UK	Retrospective audit.	To evaluate a new ID screening service within a women’s prison in England.	Of 23 women referred for secondary assessment, only 4 underwent the Hayes Ability Screening Index (HASI); others released.	Difficulty with academic work at school. Attend special needs school/class. Extra help with reading, writing, learning. Requires help with filling in forms, shopping, handling money, using public transport. Been diagnosed with learning disabilities, learning difficulties, dyslexia, attention-deficit/hyperactivity disorder (ADHD), dyspraxia.
[30] Braatveit et al. (2018) Norway	Cross-sectional study.	To validate the HASI as a screening instrument for identifying intellectual disabilities in a population of inpatients with substance use disorder (SUD) using all three ICD-10/DSM5 criteria in classifying ID as the validation criterion.	It is important to stress that the HASI is not a diagnostic instrument but is a screening tool used to detect individuals who should receive further assessment. Based on this, having a strong sensitivity and a weaker specificity is less of a problem than if the opposite were true, and the HASI is a timesaving instrument for detecting further assessment needs when considering ID, borderline intellectual disability, and borderline intellectual functioning in the SUD population.	Increased support needs, mental health problems.
[31] Braatveit et al. (2022) Norway	Cross-sectional study.	To investigate the convergent, predictive, and discriminant validity of the HASI subtest background information in identifying in-patients with SUD, mild to borderline intellectual disability (MBID) or non MBID.	Among the HASI subtests, background information was the strongest predictor and has acceptable validity for screening MBID among in-patients with SUD. To have an early indication of MBID can be of clinical value for patients with MBID because they are known to have higher treatment dropout rates and more negative treatment experiences than the general SUD in-patient group. A HASI background information cut between 6 and 7 showed a sensitivity of 78% and a specificity of 72%.	Showed greater involvement with public support systems during childhood. Reported more childhood learning difficulties. Had higher relapse rates during treatment. Were more likely to have developmental and conduct disorders.
[32] Cashin et al. (2006) Australia	Cross-sectional study.	To determine the prevalence of intellectual disability in the New South Wales inmate population.	Prevalence of ID lower than community prevalence. Positive correlations at a significance level of 0.01 between the HASI score and the overall full scaled score and the scaled scores in the verbal and performance scales.	Difficulty processing information. Learning disorders.
[33] Catalano et al. (2020a) Australia	Literature review.	Targeted review of the literature on screening tools for cognitive impairment and adaptive functioning in prisons.	From 115 journal articles, 26 tools were identified.	Educational background. Availability of social support and difficulties with arithmetic (calculation), reading and writing (spelling exercise), language comprehension and behaviour tasks.
[34] Catalano et al. (2020b) Australia	Literature review.	Targeted literature review to identify screening tools and diagnostic assessment instruments with proven or potential application in prison populations.	In total, 135 peer-reviewed articles were identified in relation to diagnostic instruments. In total, 15 assessment instruments indicated were suited to forensic settings. The use of threshold questions at prison reception is applied in several jurisdictions and can be useful. Through such a staged process, prisons reduce the number of diagnostic assessments required and, concomitantly, lessen the demands upon clinical staff.	Difficulties with schoolwork. Previously assessed at school as needing additional help. Attended a special school or a special needs class. Needed extra help with writing, reading, learning. Previously diagnosed as having a disability. Requires help now with filling in forms, shopping, handling money, using public transport. Received a disability pension, disability support services. Matches words with pictures, reasoning skills and abstract problem-solving ability.
[35] Chaplin et al. (2017) UK	Cross-sectional study.	To identify prisoners with ID and compare their characteristics with prisoners without NNDs regarding demographic profile, mental health, suicide risk, and offences.	Following screening, 18 male prisoners were identified with ID.	Being single. Homeless. Unemployed. Not having any qualifications before coming into prison. Left school before the age of 16. Problems with reading and writing.
[36] Delahunty et al. (2022) UK	Cross-sectional study.	To identify clinical features and characteristics of children with ID in a population of 6–18-year-olds in mainstream school attending paediatric developmental clinics.	The number of children that met the criteria for ID was 28 (22%). The majority of children meeting the criteria for ID were male (64.3%). Children in the ID group were more likely to have had contact with all services.	Concerns about development in early years. Learning and developmental delay. Multiple health problems in past. Undergone testing for genetic abnormalities. Mother who smoked during pregnancy. Speech and language concerns in the early years. Family history of confirmed or suspected ID. History of health problems likely to impact on development. Having a lower height centile. Parental unemployment.
[37] Dias et al. (2013) Australia	Cross-sectional study.	To estimate the prevalence of co-occurring mental disorders among prisoners with ID and explore the association between ID and mental disorder.	The positive association between ID and lifetime psychiatric diagnosis was consistent with previous studies. There is an association between prisoners with ID having higher co-occurring mental disorders at (52.5%) current (37.2%).	Attended a special school. Diagnosis of ID. Education of less than 10 years. Unemployment in last 6 months. Unstable accommodation. Previous juvenile and adult imprisonment. Social disadvantage. Psychological distress. Mental illness. Use of psychotropic medication.
[38] Donohue et al. (2014) USA	Correlational study.	To examine the utility of a brief test of intelligence (PROFOKS) in inpatients in a forensic psychiatric unit.	The PROFOKS appears to be a useful screening tool in identifying intellectual disability in a forensic psychiatric population and correlated with the Wechsler Adult Intelligence Scale (WAIS).	Low score on tool.
[39] Douglas and Cuskelly (2012) Australia	Qualitative focus group study.	To extend the understanding of police in Queensland, Australia to identify an individual as having ID.	Police officers reflected that they could identify ID by appearance and behaviours. Failure to identify people with ID resulted in lack of appropriate services. HASI screening tool was recommended.	Appearance. Language difficulties. Problems with comprehension. Inappropriate behaviour for age. Problem behaviour.
[40] Durbin et al. (2018) Canada	Cross-sectional observational study.	To assess the prevalence of borderline or lower intellectual functioning among homeless adults with mental illness and determine whether lower intellectual functioning is associated with longer lifetime duration of homelessness.	Lifetime homelessness duration was approximately three years longer, or almost twice as long, for individuals with borderline or lower intellectual functioning. Findings support existing calls to consider screening for intellectual functioning in programs that serve homeless individuals and to tailor interventions to better support this disadvantaged population.	Immigrants. Completed some high school. Receipt of disability income support. Lifetime duration of homelessness. Difficulty with routine tasks, understanding information.
[41] Fischer et al. (2014) Brazil	Literature review.	To identify the tools for identification of children with developmental disabilities and assess the psychometric properties and feasibility of the tools used in low- and middle-income countries (LMICs).	Fourteen tools used in LMICs, with three reporting adequate psychometric properties and meeting most of the feasibility criteria. Three tools appear promising for use in identifying and monitoring young children with disabilities at primary healthcare level. They varied significantly in their psychometric performance and feasibility. None of the tools fully met all the criteria established by the experts. Four tools had good psychometric properties (over 80%). However, only three met most items of feasibility listed by the experts.	Difficulties in communication, gross motor development, fine motor development, problem solving, personal social skill, daily living skills, socialisation, cognitive, personal, social, expressive language, receptive language. Seizures. Socioemotional difficulties. Difficulties in vision and hearing, eye–hand coordination and locomotor skills, self-help skills, play, language and concept development, learning, development and behaviour, primitive reflexes, and social-personal aspects skills.
[42] Ford et al., 2008 UK	Cross-sectional study.	To gain data from a representative sample of the young offending population in the northeast UK and to determine whether the HASI is an effective means of identifying those young people within youth justice services for ID.	The HASI was too over-inclusive; it reported a positive result (i.e., recommendation to refer for further assessment) for a considerable number of individuals who did not meet the criteria for ID when assessed using diagnostic measures commonly used in clinical practice. A screening tool is not expected to demonstrate perfect performance when compared with diagnostic criteria, as it is not a substitute for full assessment. The HASI is designed to be over-inclusive; however, the HASI appears to be excessively sensitive, resulting in an unacceptably low specificity to ID.	School exclusion. Behavioural disturbance in mainstream school. Reading difficulties. Needing special help.
[43] García-Largo et al., 2020 Spain	Literature review.	To provide a global overview of the available evidence regarding ID rates in prisons.	The prevalence of ID among prison inmates is high and is much higher than in the general population.	Low scores. Less capable of understanding their rights. Problems in understanding information. More likely to suffer harassment and segregation, to have more depressive and anxiety symptomatology, higher risk of social isolation and exclusion from activities.
[44] Gendle and Woodhams, 2005 UK	Qualitative interviews.	To explore police officers’ perceptions of suspects with ID, their knowledge and understanding of ID, how well they apply procedures (e.g., use of appropriate adults) when interacting with such individuals, the adequacy of training received on ID-related issues.	Officers often confused ID with mental illness. Officers expressed concern over failing to recognise ID, especially in suspects who do not display visible indicators. Officers recognised difficulty in applying the law fairly when suspects did not understand cautions or procedures. Officers noted problems communicating with suspects with ID. Many were unsure of how to phrase questions without leading or coercing. Training was often theoretical, lacking practical, experiential components. Officers wanted more hands-on training and clearer guidance on identifying and supporting people with ID. Officers generally expressed empathy and a desire to do the right thing but felt underprepared and unsupported by existing systems.	Easily influenced by people with a bit more common sense. Engaging in minor crime, robbery, assault. Cannot read. Difficult to understand.
[45] Grigorenko et al., 2015 USA	Cross-sectional observational study.	To assess the academic skills (reading and math) of juvenile detainees in Connecticut, identify the prevalence of learning disabilities using performance-based screeners, identify academic deficiencies that could relate to learning or intellectual disabilities, provide baseline data.	Estimate that at least 10% to 15% of juveniles in detention (i.e., at least twice the rate in the general population) have various forms of severe learning disabilities, often comorbid with other developmental and neuropsychiatric conditions, particularly ADHD. Yet these estimations have not been substantiated in large-scale studies.	Difficulties in academic skills of reading, spelling, math.
[46] Hayes et al., 2007 UK	Cross-sectional, observational prevalence study.	To determine the prevalence of ID among inmates in a major UK prison using both standardized intelligence and adaptive behaviour assessments.	The results indicate that the prevalence of ID is higher than previous research in the UK has indicated.	Communication difficulties. Receive poor healthcare.
[47] Hayes, 2002 Australia	Cross-sectional study.	To compare the Kaufman Brief Intelligence Test (KBIT) and the Vineland Adaptive Behavior Scale (VABS) with the HASI.	The HASI was effective in identifying those prisoners who need full-scale diagnostic assessment. The HASI is designed to be administered by non-psychologists. The index does not diagnose the presence of ID, but rather, identifies those people who either need to be referred for further diagnostic assessment or, in a police setting, need to have implemented the protections offered to vulnerable suspects.	Low score on tool.
[48] Kaal et al., 2015 Netherlands	Mixed-method pilot implementation study.	To pilot the Screener for Intelligence and Learning disabilities (SCIL) in detention settings to determine the feasibility of screening for ID in prisons, the added value for staff of knowing whether a detainee has ID, the prevalence and characteristics of ID.	The SCIL can be successfully implemented within the criminal justice system (CJS), especially when prison workers determine their own ways in which they fit the screening in their daily routines and when they receive adequate instructions on how to administer the instrument.	Difficulties with calculations, writing a simple line without errors, reading, drawing time on a clock, elementary skills needed in daily life.
[49] Leung et al., 2013 China	Psychometric validation study.	To evaluate the reliability and validity of the cognitive subtest of the Preschool Developmental Assessment Scale (PDAS) for Chinese children.	The cognitive subtest of the PDAS was found to be a promising screening tool for the identification of preschool children with developmental disabilities.	Low scores on areas within tool.
[50] Mahmood, et al., 2021 USA	Cross-sectional observational study.	To examine rates of cognitive and functional impairment and the association between cognitive performance and functional capacity of sheltered adults experiencing homelessness.	A total of 65% scored in the cognitively impaired range on a brief cognitive screening test, 30% had impaired processing speed, and 11% met cognitive criteria for ID.	Homelessness. Reading, language, math deficiencies.
[51] Mason and Murphy, 2002 UK	Psychometric development and validation study.	To develop a screening tool to detect those who may have ID or borderline ID (BID) in the CJS and create a tool that probation officers can easily administer without clinical training.	The screening measure has a good level of sensitivity but somewhat lower specificity (as for most screening tools). The assessment of cognitive functioning requires careful consideration of cultural, ethnic, and socioeconomic factors. Almost by definition, screening tools ignore these.	Had a learning disability. Been to special school. Received help for a mental health problem. Live with relatives. Problems with everyday coping skills. Difficulties with verbal functioning. Ability to keep appointments, follow rules, control feelings.
[52] McCarthy et al., 2016 UK	Cross-sectional descriptive study.	To identify prisoners with NDD, including ADHD, autism spectrum disorder (ASD), and ID, and to compare characteristics (sociodemographic, educational, criminal history) of prisoners with and without NDD.	The study identified 87 prisoners who screened positive for one or more types of NDD. Participants with NDD were significantly younger and more likely to be single [(odds ratio) OR = 2.1], homeless (OR = 3.4) or unemployed (OR = 2.6) before they came into prison. They also had poorer educational achievements than those without NDD. Over 80% of those with NDD had a previous conviction or imprisonment.	Single. Homeless. Unemployed. Poorer educational achievements. Self-reported difficulties with attention, impulsivity, hyperactivity, social communication, behaviour, literacy, functioning.
[53] McCarthy et al., 2015 UK	Cross-sectional screening and diagnostic assessment study.	To identify prisoners with NDDs to determine the best approach to the recognition and assessment of individuals with NDDs within the UK prison system.	The study identified 24 participants who met the criteria for mild/borderline ID, 3 with an estimated IQ of less than 70, and 21 with borderline intellectual functioning (IQ of 70–84); 87 prisoners with NDD, 79 screened and 8 self-reported a diagnosis; 65 screened positive for ADHD, 46 screened positive for ASD, and 33 screened positive for ID. Most of those with NDD (51 percent) had previously gone unrecognised. Providing access to skilled, standardised assessment can result in increased recognition and identification of NDD.	An existing diagnosis or previously suspected. Low scores. Less likely to read, write. Experienced homelessness.
[54] McKenzie et al., 2012a UK	Psychometric validation study.	To assess the validity of an intellectual disability screening tool, the Learning Disability Screening Questionnaire (LDSQ).	Using the LDSQ cut-off score obtained from the original community standardisation sample gave sensitivity of 82.3% and specificity of 87.5%. This compares with 91.2% and 87%, respectively, for the LDSQ as used with the original community sample.	LDSQ cut-off score.
[55] McKenzie et al., 2012b UK	Pilot psychometric validation study.	To evaluate aspects of the validity of the Child and Adolescent Intellectual Disability Screening Questionnaire (CAIDS-Q) as a screening tool for young offenders with ID.	Good internal consistency was indicated by a Cronbach’s alpha value of 0.723. A significant difference was found between the CAIDS-Q scores of those with (mean ¼ 37.9, SD ¼ 18.1) and without ID (mean ¼ 84.7, SD ¼ 12.8), using an independent *t*-test.	Low scores on tool.
[56] McKenzie et al., 2019 UK	Pilot feasibility and validation study.	To evaluate the use of the LDSQ in a homeless service.	Three people were indicated by LDSQ scores as likely to have ID. Two subsequently had this status confirmed, but the third did not attend for further assessment. All but two items on the LDSQ showed complete agreement. High and significant agreement was found between raters for LDSQ total and percentage scores.	Help needed with spelling. Could use only digital clock to tell time. Prior special school attendance. Difficulties with telling time, writing, and reading. Co-occurring issues: mental health problems, substance misuse, previous offending, and physical health concerns.
[17] McKenzie et al., 2023 UK	Correlational validation study.	To examine the concordance between the LDSQ and assessments of ID (intellectual and adaptive functioning and developmental history) and inter-rater reliability.	The LDSQ was found to have good inter-rater reliability, with significant agreement between total percentage LDSQ scores for staff-completed and self-completed questionnaires. Some discrepancy in LDSQ scores for some people who were indicated as likely to meet the criteria for ID. The LDSQ, when completed by staff, had levels of accuracy that are consistent with those found in other settings.	Low score on tool.
[57] McKinnon et al., 2015 UK	Multi-part prospective descriptive study.	To ascertain the efficacy of current police reception screening to detect detainees with ID.	The rate of detainees with suspected ID was 2–3 percent. The standard police screen detected 25 percent of these detainees in part 1. When the new screen was introduced in part 2, the sensitivity for ID increased to 83 percent.	Learning or educational problems. Ability to read. Low scores.
[58] Murphy et al., 2017 UK	Prospective observational feasibility study.	To assess the feasibility and the utility of screening for ID using the LDSQ.	Of the total 2429 who were screened, 169 were identified as being more likely to have ID on the LDSQ (7%), and the remainder were considered unlikely to have ID.	Dyslexia. Low score on tool.
[59] Nieuwenhuis et al., 2022a Netherlands	Cross-sectional observational study.	To establish the association between aggressive behaviour and mild ID (MID)/borderline intellectual functioning (BIF) among psychiatric patients.	A total of 239 (20.4%) showed a SCIL score of 15 and lower.	Low score on tool. Poor impulse control. Difficulty coping with complex social environments. Higher prevalence of psychiatric comorbidities.
[60] Nieuwenhuis et al., 2022b Netherlands	Observational within-subject longitudinal study.	To examine the influence of the severity of psychiatric symptoms on the SCIL scores.	A total of 86% of patients received the same SCIL classification in terms of screening positive/negative for MID/BIF, regardless of the administration time. BIF was classified in 76% as the same, while MID was the same in 86%. No BIF or MID was the same in 86% of cases.	Involuntary admission. Subject to coercion. More aggression, physical aggression. Experience of sexual abuse. PTSD not recognised (person unaware).
[61] Nieuwenhuis et al., 2021 Netherlands	Cross-sectional observational study.	To establish the prevalence of ID in different mental healthcare settings and estimate the percentage of cognitive decline.	From screening 1213 persons, 502 (41.4%) were identified as borderline ID.	Low score on tool. Long history of psychiatric care. Social functioning problems for at least two years. Lower quality of life. Poorer functioning. Low intellectual functioning.
[62] Nieuwenhuis et al., 2017 Netherlands	Prospective dynamic cohort study.	To determine the percentage of MID/BIF among admitted psychiatric patients according to the SCIL.	Of the 208 patients who were screened with the SCIL, 91 (43.8%) were found to be SCIL positive.	Conduct problems. Social withdrawal. Substance abuse. Reduced coping skills. Easily react with verbal aggression. Refusal behaviour.
[63] Nijman et al., 2018 Netherlands	Psychometric validation study.	To develop and validate a brief, reliable, and valid screening tool (SCIL) for identifying MBID.	The validity of using the SCIL for the youngest respondents of 12 and 13 years, also for detecting an IQ of below 70, turned out to be lower when compared with the results with the older juveniles or adults. This underlines that it is not advisable to use the SCIL in minors under the age of 14 years. T-tests revealed no significant differences in SCIL scores between boys and girls.	Poor cognitive ability. Poor mathematical skills, writing skills. Poor social and adoptive functioning.
[64] Pratt, 2015 USA	Meta-analytic review (dissertation).	To examine intellectual deficits in persons who are homeless.	Estimated prevalence among the homeless population: ID (IQ < 70): 8.9%, BID (IQ 70–85): 23.2%.	Homelessness. Increased risk of exploitation. Having a higher likelihood of being denied rights and opportunities. Prevalence of at least one comorbid mental disorder. Difficulty securing and maintaining employment.
[65] Ramsay, et al., 2020 UK	Qualitative exploratory study.	Examine staff views of the usefulness of the Learning Screening Tool (LST) and Adaptive Functioning Checklist—Revised (AFC-R) on offending behaviour programme selection.	The application of clinical judgement was a strong theme, with a clear view that the screening tools are most effective when clinical judgement and interpretation is applied alongside the screening tools. A cut-off of three was used to identify people who may be more likely to have ID and/or learning challenges.	Issues with social functioning, communication, social participation, personal independence, functioning in activities, work skills, education. No fixed abode.
[66] Ross et al., 2020 UK	Psychometric validation study.	To investigate the psychometric properties of the Adaptive Functioning Assessment Tool (AFAT).	The AFAT is both a reliable and valid measure of adaptive functioning (AF) in a prison environment.	Issues with communication, socialisation, independence, functioning at work, functioning at education. Poor hygiene or daily living skills. Inability to manage money. Limited participation in education or work programmes.
[67] Simonoff et al., 2006 UK	Population-based cross-sectional study.	To estimate the prevalence of mild ID in a UK urban population and examine the factors that relate to educational identification.	Only 15% of those with IQ < 70 had a statement of special educational needs or attended a school for moderate learning difficulties.	Low score on tool. Low academic performance. Social communication difficulties.
[68] Søndenaa et al., 2010 Norway	Cross-sectional validation study.	To explore methods for better identification of ID at an early stage during criminal proceedings.	A total of 10.8% (n = 15) of the participants showed an IQ below 70, and an additional 12.2% (n = 17) had scores in the borderline range (IQ 70–79). A total of 23.0% had considerable intellectual impairments, defined by an IQ below 80 as measured by the Wechsler Abbreviated Scale of Intelligence (WASI). Out of the 46 cases referred for neuropsychological examination, 15 scored below IQ 70 on the WASI, 14 scored between IQ 70–79, and 18 scored above IQ 80. HASI scores below 85 also require further assessments.	Low score on tool.
[69] Søndenaa et al., 2008 Norway	Cross-sectional observational study.	To calculate the prevalence of inmates with ID and identify historical, medical, and criminological characteristics.	Two-thirds (10 of 15) of the individuals with ID were identified, and only 16 of the 124 individuals without ID were wrongly identified.	History of truancy. Need help in school. Dyslexia. ADHD. Having a learning disability. History of special educational needs. Repeated incarcerations.
[70] Stewart et al., 2016 Canada	Cross-sectional observational study.	To estimate the prevalence of cognitive deficits among a sample of incoming male federal offenders.	Education level and the presence of a potential learning disability were significantly related to cognitive deficits. Lower educational achievement was associated with having either moderate or severe cognitive deficits. Offenders with cognitive deficits were three times more likely to have a learning disability than offenders with no deficits.	Education level. Lower educational achievement. Unstable employment history. Alcohol dependence.
[71] To et al., 2015 Netherlands	Validation study.	To assess the validity of the Dutch version of the HASI in persons with a substance abuse problem residing in mental health services.	A significant positive relationship was found between the full-scale IQ of the WAIS-III and the HASI score, indicating that the higher the IQ score of a person, the higher the HASI score will be. This finding is congruent with results from previous studies.	Substance abuse. Mental health problems.
[72] Tort et al., 2016 Spain	Cross-sectional descriptive epidemiological study.	To determine the prevalence of ID and BIF in the Spanish prison population and the prevalence of ID in prison psychiatric units and hospitals.	Prevalence of 3.77% for IQ under 70 among inmates to whom the TONI-2 test was administered.	Behavioural disorder. Difficulty understanding prison rules. Poor impulse control.
[73] Tzouriadou et al., 2021 Greece	Cross-sectional observational validation study.	To investigate if teachers’ judgements with the use of an appropriate screening tool can detect Roma students at risk for developmental disabilities and predict their actual language achievement.	A total of 23 students with low scores were seen as at risk of ID. A high proportion of both Roma and non-Roma students were at risk for developmental disabilities based on teacher screening.	Score low on tool.
[74] Uzieblo et al., 2012 Belgium	Theoretical and critical review.	To discuss the incorporation and possible implications of using the Cattell–Horn–Carroll (CHC) model of intelligence in the CJS.	The prevalence of intellectual disability is not overrepresented in the criminal justice system but higher rates in mental health.	Low intelligence. Adaptive functioning.
[75] van Esch et al., 2020 Netherlands	Cross-sectional psychometric validation study.	To investigate the psychometric properties of the SCIL in a population of mentally ill Dutch detainees.	The SCIL gives a quick and accurate indication of whether a person is at risk for ID. Although both the reliability and validity of the SCIL are lower in the study population than in regular prison populations, all psychometric properties could be classified as acceptable for the SCIL.	Limited social network. Experience problems in maintaining contact with their family.
[76] Vella, 2014 USA	Cross-sectional observational study.	To examine the level of cognitive and functional impairment in a sheltered homeless population and the effects of impairment on service utilisation.	Significant cognitive and functional impairment was observed. Most participants performed in the average range for IQ and general cognition, but many showed low average or below average performance in key functional areas. No significant link was found between cognitive/functional scores and housing or service outcomes. Important to routinely conduct cognitive screening in shelters.	Low score on tool. Issues with reading, vocabulary, reasoning, functional capacity.
[77] Verheijen et al., 2022 Netherlands	Cross-sectional observational study.	To investigate MBID prevalence and its relationship with psychopathology and self-sufficiency problems among young adult violent repeat offenders.	A total of 240 participants (55.6%) were assessed using the SCIL. The prevalence of MBID in a group of young adult violent repeat offenders, obtained with a screening instrument for MBID, was 51.1%.	Low scores on tool. Poor adaptive functioning
[78] Wakeling and Ramsay, 2020 UK	Validation study.	To validate the LST and the AFC-R.	Regardless of the administration time, 86% of patients received the same SCIL classification (in terms of screening positive/negative for MID/BIF). Need for better adherence to guidance and training for staff in tool administration and interpretation.	Poorer adaptive functioning. Challenges in social participation and daily activities. Socio-environmental risk factors (e.g., homelessness, lack of education).
[79] Young et al., 2018 UK	Cross-sectional observational study.	To identify the prevalence of NDDs, specifically ADHD, ASD and ID, in a prison population and explore their comorbidity with psychiatric symptoms.	Nine percent (n = 35) identified with ID. Inmates with ID had significantly higher scores on both DBSP subscales compared with those without ID. Forty percent of the ID group met criteria for ADHD.	Low score on the tool. Destructive behaviour. Social problems. Sought attention from staff.
[80] Young et al., 2013 UK	Cross-sectional observational screening and diagnostic study.	To assess the prevalence of ID and ADHD among individuals detained by police and assess the effectiveness of current police risk assessment procedures in identifying these vulnerabilities and prompting use of appropriate adults during interviews.	Of these individuals, the current study identified that 7 people screened positive for ID and 24 screened positive for current ADHD. However, only two (28.6%) and one (4.2%) had an appropriate adult, respectively.	Self-harm. Mental health problems. Substance use. Need help with reading, writing.

**Abbreviations**—**ADHD**—attention-deficit/hyperactivity disorder; **AF**—adaptive functioning; **AFAT**—Adaptive Functioning Assessment Tool; **AFC-R**—Adaptive Functioning Checklist—Revised; **ASD**—autism spectrum disorder; **BID**—borderline intellectual disability; **BIF**—borderline intellectual functioning; **CAIDS-Q**—Child and Adolescent Intellectual Disability Screening Questionnaire; **CHC**—Cattell–Horn–Carroll (model of intelligence); **CJS**—criminal justice system; **GAF**—Global Assessment of Functioning; **HASI**—Hayes Ability Screening Index; **ID**—intellectual disability; **IQ**—intelligence quotient; **KBIT**—Kaufman Brief Intelligence Test; **LDSQ**—Learning Disability Screening Questionnaire; **LMICs**—low- and middle-income countries; **LST**—Learning Screening Tool; **MBID**—mild to borderline intellectual disability; **NDD/s**—neurodevelopmental disorder/s; **PDAS**—Preschool Developmental Assessment Scale; **PROFOKS**—knowledge of PROverbs, Fund of Knowledge and Similarities; **RAPID**—rapid assessment of potential intellectual disability; **SCIL**—Screener for Intelligence and Learning Disabilities; **SSM-D**—Self-Sufficiency Matrix—Dutch version; **SUD**—substance use disorder; **TONI-2**—Test of Nonverbal Intelligence; **UK**—United Kingdom; **UPSA-B**—UCSD Performance-Based Skills Assessment—Brief; **VABS**—Vineland Adaptive Behavior Scales; **WAIS**—Wechsler Adult Intelligence Scale; **WASI**—Wechsler Abbreviated Scale of Intelligence.

## 3. Results

The findings present the study characteristics, and the key questions identified (1) what indicators currently exist for intellectual disability when no formal diagnosis is present, (2) what screening tools are currently in use to screen for intellectual disability when no formal diagnosis is present, (3) who administers these tools, and (4) are there particular marginalised groups identified as being at risk.

### 3.1. Study Characteristics

The studies were conducted in a variety of settings: criminal justice settings (n = 31); mental health services (n = 8); homelessness (n = 6); combined criminal justice and mental health services (n = 4); primary healthcare (n = 1); and a multi-site study (n = 1) incorporating social workplaces, probation services, and organisations that provide treatment facilities for addiction. Study designs included quantitative (n = 46), qualitative (n = 2), mixed methods (n = 1), literature reviews (n = 5), dissertations (n = 2), and discussion papers (n = 1). Furthermore, 51 studies were conducted with the adult population and the remaining 6 with a child population. Research was primarily conducted in the UK (22 studies) followed by The Netherlands (9 studies); Australia (7 studies); USA (5 studies); Norway (4 studies); Spain and Canada, 2 studies each; and 1 study each from Sweden, Brazil, China, Belgium, Greece, and Jordan.

### 3.2. What Indicators Currently Exist for Intellectual Disability When No Formal Diagnosis Is Present?

Indicators of intellectual disability were identified across (1) educational (preschool, primary, and secondary school) settings, (2) social care services such as homelessness and community services, and (3) criminal services such as courts, probation, and prison services. These indicators are related to experiences, behaviours, difficulties, and observations (Table 4).

### 3.3. What Screening Tools Are Currently in Use to Screen for Intellectual Disability When No Formal Diagnosis Is Present?

A total of 42 studies utilised various screening tools, and these tools, their descriptions, and studies employing them are presented in Table 5.

### 3.4. Who Administers These Tools

The review identifies a diverse range of individuals who administered assessment tools across different settings. Within the criminal justice system, these tools were predominantly administered by research teams (n = 12) and healthcare professionals (n = 6). Additionally, research teams in other settings also administered the tools (n = 5), making research teams (n = 17) the largest group overall. In homeless services, both trained (n = 1) and untrained staff (n = 3) administered some tools. Healthcare professionals (n = 5) and trained staff (n = 1) were involved in mental healthcare settings. A notable finding is the wide variety of personnel and locations conducting these screenings. The dominance of research teams in administering these tools may reflect resource limitations or lack of training in operational settings. This raises questions about how best to embed screening into routine clinical workflows, particularly where specialist training is unavailable.

### 3.5. Particular Marginalised Groups Identified as Being at Risk

A recurring theme across the reviewed literature is the disproportionate focus on male populations within both the criminal justice and homelessness contexts, often tied to lower-level offences. The limited representation of female participants in these studies suggests a significant gap in understanding the full scope of intellectual disability across genders. Most studies did not extensively address specific marginalised groups. However, McCarthy et al. [53] in the UK found that people from Black or minority ethnic backgrounds are particularly at risk of unrecognised neurodevelopmental delays potentially indicating intellectual disability. This may be due to culturally insensitive assessment tools and referral biases favouring the identification of intellectual disability in White prisoners. The review underlines the strong connection between mental health issues and the criminal justice and homelessness services, with these populations being at high risk. Two studies called for better education among healthcare professionals regarding the comorbidity of mental health and intellectual disability [25,40]. Sociodemographic factors such as immigrant background are significant risk factors in homelessness services, and people with borderline or lower intellectual functioning were found to experience prolonged or lifelong homelessness [40]. Additionally, very few studies examined screening indicators among female populations or diverse ethnic groups, pointing to a critical evidence gap. This underrepresentation must be addressed to develop inclusive and equitable screening strategies.

## 4. Discussion

The findings suggest that intellectual disability can be identified through patterns of personal experiences, observable behaviours, functional challenges, and subjective professional observations across educational, social care, and criminal justice settings. Common indicators include difficulties in communication, comprehension, safety awareness, and academic performance, along with socially vulnerable behaviours often identified by professionals or carers. These patterns were evident in educational settings (preschool through secondary school), homelessness services, and within the criminal justice system.

A diverse range of tools has been utilized to identify intellectual disability without a formal diagnosis across multiple sectors. Most tools were employed within criminal justice settings, with research teams (n = 17) being the primary group conducting assessments, followed by healthcare professionals (n = 6). Within homelessness services, both trained (n = 1) and untrained staff (n = 3) also administered screenings. These variations indicate the lack of a standardised approach, emphasising the need for a professionalised system wherein qualified healthcare staff oversee screenings to ensure consistency and accuracy in identifying intellectual disability. A recurring theme across the screening tools reviewed is their focus on adaptive behaviour, such as difficulties with communication, daily living skills, and social functioning. This aligns with existing definitions of intellectual disability, which require deficits in both intellectual functioning and adaptive behaviour. There is a relationship between IQ and adaptive behaviour within intellectual disability populations, reinforcing the value of screening tools that assess adaptive functioning, and such tools warrant further exploration for broad-based screening.

This review highlights the need for identifying individuals with undiagnosed intellectual disabilities and having an assessment tool based on identified indicators that can be used across different settings. However, such a tool needs to be validated by a longitudinal study to evaluate its effectiveness that incorporates Patient and Public Involvement (PPI) and includes various marginalised populations such as homeless individuals, substance abusers, migrants, international protection applicants (IPA), and those involved in criminal activities to understand their specific challenges and indicators [81]. In addition, consideration needs to be given to the effectiveness of healthcare professionals’ educational programmes regarding awareness of, recognising, and assessing intellectual disability [82,83].

The robustness of tools like HASI, SCIL, and LDSQ is supported by acceptable sensitivity and specificity in research settings; however, their cultural adaptability across populations remains insufficiently validated and requires further study. Future directions may include AI-enhanced or digital screening platforms to improve accessibility and consistency in identification, especially in under-resourced settings.

Culturally sensitive and contextually appropriate screening instruments are needed to accommodate varying needs and to ensure accurate identification, especially among marginalised populations. Screening tools should be validated across diverse settings to avoid biases in identifying intellectual disability. The review identified several marginalised groups at risk, with most studies focussing on men involved in minor criminal offences and the homeless. These findings highlight the need for tailored approaches in screening and supporting individuals with intellectual disability across various settings, particularly among marginalised and high-risk groups. The involvement of various personnel, predominantly research teams, highlights the need for a broader focus on screening and a professional group to accept responsibility for screening.

Undiagnosed intellectual disability can profoundly affect individuals and their families, necessitating a comprehensive approach to address the issue. This involves raising awareness, ensuring access to diagnostic services, fostering cultural sensitivity, and enacting strong policy support. Early diagnosis and intervention are essential for enhancing outcomes and enabling individuals with intellectual disabilities to lead fulfilling lives [84,85]. Without a diagnosis, individuals may miss out on necessary medical and psychological support or fail to access social services designed to improve their quality of life and independence [86]. They might also face social stigma and misunderstanding, often mislabelled as lazy or uncooperative. This lack of understanding can lead to low self-esteem and mental health issues [87,88]. Consequently, individuals may exhibit challenging behaviours as a coping mechanism, which can be misinterpreted and poorly managed without a proper diagnosis [89]. This situation often results in a revolving door system in health and social care, where individuals with undiagnosed intellectual disabilities present with complex medical and psychological needs [90]. They may frequently visit healthcare facilities seeking treatment for misunderstood symptoms, leading to increased utilisation of primary care, emergency services, and specialised medical consultations [91,92]. Therefore, early detection and diagnosis of intellectual disability are crucial for providing timely and appropriate interventions. Recognising and addressing the signs early allows for better support and resources tailored to the individual’s needs, significantly improving their outcomes and quality of life [93].

Identifying all individuals in a community who meet the criteria for low IQ and deficits in adaptive skills is challenging. It requires extensive assessments of potentially affected individuals in both areas, which takes several hours per person and requires their consent and cooperation [94]. Despite these challenges, it is crucial to determine the proportion of people with mild or borderline intellectual disability to ensure they receive adequate and appropriate services and support. The review noted that studies from various countries demonstrate a global interest in understanding and screening for undiagnosed intellectual disability in different contexts.

The review highlights the concept of “intuitive” knowing, which involves immediate understanding based on feelings rather than factual evidence. This intuitive knowledge plays a crucial role alongside evidence-based practice, as it enhances care by identifying subtle cues and patterns in individuals’ behaviours and conditions [95,96]. However, the review notes a gap regarding specific steps to take when suspecting an undiagnosed intellectual disability. This omission is concerning, as it neglects to address vulnerabilities and individuals’ specific health, social, and cognitive needs, which are crucial under the United Nations Convention on the Rights of Persons with Disabilities [97].

To address and enhance our understanding and improve practices in identifying and supporting individuals with intellectual disabilities who have not received a formal diagnosis, there needs to be a screening policy at a national level mandating screening protocols in health, education, and social care settings to detect potential intellectual disabilities. A key professional who can address these issues is an intellectual disability nurse. While intellectual disability nurses are well positioned to play a central role in screening and support, this profession is not universally recognised across all countries. In contexts where specialist intellectual disability nursing does not exist, we recommend that core competencies in recognising and responding to intellectual disability be embedded into health and social care curricula and required as part of continuing professional development. Equipping health and social care professionals with foundational training in intellectual disability can help ensure consistency and quality in early identification efforts across diverse healthcare systems.

Where intellectual disability nurses exist, they are in prime positions, as they are educated to identify signs of intellectual disability through comprehensive assessments of developmental history, behaviour, social interactions, and cognitive functioning. Intellectual disability nurses bring specific competencies in communication, behavioural interpretation, interdisciplinary collaboration, and long-term care planning, which make them uniquely suited for identifying and supporting individuals with complex needs. They work within multidisciplinary teams, sharing their findings with psychologists, psychiatrists, and educators to comprehensively understand an individual’s needs. They communicate closely with families and caregivers, providing essential information, education, and support. These nurses advocate for early identification and appropriate referrals, ensuring individuals receive appropriate care and resources. Their role is crucial in raising awareness, improving diagnosis, and supporting the ongoing development and well-being of individuals with intellectual disabilities [98]. Furthermore, nursing indicators are highlighted as essential tools for assessing and improving care quality in intellectual disability nursing. They offer objective assessments that enhance clinical practice, evaluate care quality, and support informed decision making for people receiving nursing care [99]. Nursing indicators in intellectual disability care evolve with changing care models and standards, enabling proactive and evidence-based quality care that meets the diverse needs of individuals using these services.

In countries such as the United States, the UK, and Ireland, intellectual disability is often identified through educational systems. However, this review indicates that many individuals may still reach adulthood without a formal diagnosis. These include people who were excluded from school, those with milder forms of intellectual disability that went unrecognised, and those who immigrated after school age. Moreover, diagnostic pathways differ significantly between countries, and in many regions, there is no systematic follow-up into adulthood for individuals who showed early indicators. This highlights the need for adult screening protocols beyond educational settings. While this review highlights the value of early identification, it is important to acknowledge that not all individuals benefit equally from a formal diagnosis. Labelling can carry potential risks such as stigma, loss of autonomy, or negative stereotyping, particularly in populations that are otherwise coping or “passing” within society. However, evidence shows that many undiagnosed individuals face substantial and preventable disadvantages, including unmet healthcare needs, misdiagnosis, and exclusion from vital services [9,11]. Our call for national screening strategies therefore emphasises voluntary, consent-based, and supportive identification designed to connect individuals to tailored care, not to impose classification or remove agency.

Beyond individual barriers like stigma or lack of awareness, structural and system-level impediments can hinder progression from screening to formal diagnosis. These include fragmented service systems, variable diagnostic pathways, inadequate provider training, diagnostic overshadowing, and lack of reasonable adjustments in primary care [100]. Improving inter-agency collaboration is critical to ensure that individuals flagged for potential intellectual disability do not fall through systemic gaps. Research shows that siloed mandates, inconsistent structures across regions, and limited co-training between agencies impede coordinated assessment [101,102]. Successful models use joint frameworks, multi-disciplinary teams, shared training, and formal referral pathways to support seamless transitions between sectors. The review underscores the need for stronger inter-agency collaboration to ensure individuals identified through screening are supported across settings. Current silos between education, health, social care, and justice services often lead to missed opportunities for early diagnosis and intervention. Models such as multi-disciplinary teams, shared data protocols, and joint training initiatives have shown promise in improving coordination [103]. Developing formalised referral pathways and communication frameworks can help bridge gaps and promote a more coherent diagnostic and support process for individuals with suspected intellectual disability. Beyond the individual benefits of diagnosis, undiagnosed intellectual disability poses a significant burden on public systems and society at large. Individuals with undiagnosed intellectual disability often cycle through emergency healthcare, mental health services, substance abuse treatment, and the criminal justice system without receiving coordinated support that addresses their underlying cognitive needs [4,9]. This fragmented care results in avoidable high costs to the public purse. For example, studies estimate that individuals with intellectual disability are up to seven times more likely to be incarcerated than the general population [47], and those undiagnosed may have repeated justice involvement due to misunderstood behaviours [4]. Similarly, in homelessness services, research shows that undiagnosed cognitive impairments can lead to prolonged housing instability and poor service uptake [40,64].

From an economic standpoint, the cost of late or missed diagnosis includes both direct expenditures, such as emergency interventions, special education, and justice system costs, and indirect costs including lost productivity, caregiver burden, and social exclusion [6,104]. Evidence from developmental disability research suggests that early identification and support not only improve individual outcomes but also reduce long-term service use and financial strain on healthcare and social systems [105,106]. Thus, implementing structured screening strategies for intellectual disability, particularly in high-risk populations, should be viewed as a cost-effective public health intervention. In this regard, national screening policies can be justified not only on ethical and social equity grounds but also as fiscally responsible and efficient investments in population health. A key finding of this review is the overrepresentation of male and majority ethnic groups in the existing screening literature. This highlights a critical research gap: the potential under-identification of intellectual disability in female populations and in individuals from minority ethnic backgrounds. Gender norms and cultural perceptions of ability may influence who is noticed and referred for assessment [107,108]. Future research should prioritise inclusive sampling and explore whether existing tools adequately capture the presentation of intellectual disability across diverse groups. The transition from childhood to adulthood presents a known point of discontinuity in intellectual disability services, with many individuals falling through the gap due to age-based eligibility or service fragmentation [109]. Our review identified limited literature on how indicators or screening tools operate during this transitional period. Given that diagnostic needs and expressions of support may shift during adolescence and young adulthood, further research is warranted to ensure the continuity and appropriateness of screening during this phase.

While this review included tools such as HASI, SCIL, and LDSQ, the discussion of their psychometric robustness and cultural adaptability requires elaboration. Evidence suggests that while these tools demonstrate reasonable sensitivity and specificity within their validation populations, few have undergone rigorous cross-cultural validation or large-scale field testing with non-specialist staff. This limits generalisability. Widespread use of these instruments will depend not only on accuracy but also ease of administration, linguistic flexibility, and contextual adaptability.

A socio-ecological model provides a useful lens to understand how individual indicators (e.g., behavioural or cognitive patterns) are influenced by broader systemic factors including family, community, and service structures. This aligns with global frameworks like the World Health Organisation’s International Classification of Functioning, Disability and Health. Our findings align with a socio-ecological model of health and disability, which conceptualizes outcomes as the result of dynamic interactions across multiple levels: individual, interpersonal, community, institutional, and policy [110,111]. For individuals with undiagnosed intellectual disabilities, indicators such as educational exclusion or difficulty navigating services emerge at the individual or interpersonal level but are shaped and sustained by broader structural conditions such as fragmented care pathways, inconsistent diagnostic standards, or lack of trained professionals. A socio-ecological lens helps contextualize these barriers and underscores the need for multi-level interventions, including cross-sectoral screening strategies and inclusive policy frameworks.

Our findings also resonate with several conceptual frameworks relevant to understanding the consequences of undiagnosed intellectual disability. The concept of developmental pathways [112] highlights how early experiences, including delayed identification, can significantly influence an individual’s cognitive, social, and functional trajectory. Indicators of potential intellectual disability in childhood, if overlooked, may lead to compounding disadvantages in education, employment, and social inclusion later in life. Additionally, a social constructionist perspective invites reflection on how intellectual disability is recognised across cultural and institutional contexts [113]. Professional awareness, cultural norms, and diagnostic thresholds differ across settings and may contribute to under-identification in certain ethnic or marginalised groups, a theme that emerged in our review. Finally, labelling theory [114,115] underscores the double-edged nature of diagnosis. While receiving a diagnosis can open access to services and supports, it may also bring risks of stigma or exclusion. Our review emphasises the importance of consent-based, person-centred approaches to screening and classification to mitigate these risks while maximising benefit.

### Limitations

While this review uses a methodological framework and reporting guideline, no quality appraisal was conducted, as this review focussed on mapping the evidence. Additionally, this review focussed solely on the identification phase and did not explore the post-screening journey from referral to formal diagnosis and access to support, which should be a priority for future research. Future systematic reviews on this topic should apply rigorous inclusion criteria and quality appraisal to ensure methodological quality. Thus, this paper offers only a descriptive account of the available information. Only four databases were searched, which can be a limitation regarding the inclusion and exclusion of low- and middle-income countries. Only studies available in English and published from 2000 were included. These criteria may have limited the inclusion of non-English papers and led to underrepresentation from developing countries. It must be acknowledged that the criteria and definitions for intellectual disability can vary significantly between studies, regions, and over time. This variability can lead to inconsistencies in identifying and categorising undiagnosed cases.

## 5. Conclusions

In conclusion, this review identified several screening tools used in different countries to identify intellectual disability. In addition, this review highlighted the significant role of early identification and diagnosis of intellectual disability across various settings, particularly within marginalised and high-risk populations. The findings propose the necessity for a comprehensive screening tool validated through longitudinal studies and the importance of national screening policies. Where available, registered intellectual disability nurses play a vital role in identifying and supporting individuals with undiagnosed intellectual disabilities. Their specialised skills, combined with a collaborative and advocacy-focussed approach, are crucial in ensuring that these individuals receive timely diagnosis, appropriate support, and improved outcomes, ultimately enhancing their quality of life. In countries where this specialised role does not exist, the integration of intellectual disability education within health and social care training is crucial to ensuring equitable and informed care. This review highlights emerging systemic barriers such as poor interagency communication and service fragmentation. Notably, young people transitioning between child and adult services represent a particularly under-researched group, where screening needs and indicators may differ or be inconsistently recognised.

## Figures and Tables

**Table 1:** Search terms.

S1	“intellectual disabilit*” OR “learning disabilit*” OR “developmental disabilit*” OR “mental retardation” OR “intellectual and developmental disabilit*” OR “mental* Disabled Person*” or “mental* disab*” or retard* or “mental retard*” or “intellectual* impair*” or subnormal* or “sub-normal*” or “feeble-mind*” or “learning disorder*” OR “intellectual developmental disorder” OR neurodivers* OR “borderline” OR “cognitive impairment*” OR “cognitive disabilit*” OR “adaptive behavio#r*” OR “developmental disorder*”
S2	“Screen*” OR “Tool*” OR “Assessment*” OR “indicator*” OR “nursing indicator*” OR “clinical presentation*” OR “Screening Questionnaire” OR “screening index” OR “undiagnosed” OR “intelligent quotient” OR “skills test*” OR “screening measur*” Or “adaptive functioning” Or “nursing metric*” OR “nursing scale*”
S3	“educational difficulties” OR “Learning difficulties” OR “academic performance” OR “communication difficulties” OR “unemployment” OR “poverty” OR “mental health” OR “behavio#rs that challenge” OR “behavio#rs of concern” OR “behavio#ral patterns” OR “substance abuse” OR “homelessness” OR “prison” OR “offending behavio#r*” OR “unplanned pregnancy” OR “parenting difficulties” OR “exploitation” OR “offender*” OR “detention” OR “addiction” OR “drug abuse”
S4	S1 AND S2 AND S3

**Table 2:** Inclusion and exclusion criteria.

Inclusion	Papers that focus on people who present with functioning below that of the general population in both intellectual functioning and adaptive behaviour manifested in conceptual, social, and practical skill deficits considered typical of the individual’s peers and culture. Published primary and secondary research papers including reviews, qualitative, quantitative, and mixed-methods study designs. Grey literature including national, international guidelines, expert opinions, or reports. Papers published between 1 January 2000 and March 2024. English language papers.
Exclusion	Cognitive impairment due to chronic or acquired conditions. Papers that focus on clinical treatments. Papers that focus on health screening. Papers published before 1 January 2000. Non-English papers.

**Table 4:** Indicators of intellectual disability.

**Experiences as potentially indicative of intellectual disability**	
Developmental delay in early years	Health problems in childhood
Delay in developing fine and motor skills in early years	Socially disadvantaged or excluded
Learning challenges during childhood	Mental health problems
A learning difficulty such as dyslexia, ADHD, or autism	Personal care and daily functioning challenges
Attended “special school” or had an assistant in mainstream school	Low scores in previous screening tools
**Behaviours as potentially indicative of intellectual disability**	
Poor academic outputs	Displays maladaptive behaviours
Social skills deficits	Inappropriate relationships
Demeanour [overly friendly or age inappropriate]	Inappropriate behaviours
Executive functioning problems	Substance abuse
Aggression and conduct problems	Criminal behaviours [minor robbery and assault]
**Difficulties as potentially indicative of intellectual disability**	
Completing school	Daily functioning tasks
Communicating	Financial matters
Comprehension	Coping skills
Routine tasks [e.g., household/transport]	Securing and maintaining employment
Planning future tasks	Making positive and respectful relationships
**Observations as potentially indicative of intellectual disability**	
Abnormal movements or postures	Unexpected responses
Requires “additional time” for activities	Accommodation challenges
Extra support needs	Poor support network
Increased risk for involuntary admission, exploitation/abuse	Likelihood of being denied rights and opportunities

**Table 5:** Screening tools.

Screening Tool	Description	Studies	Psychometric Properties
Hayes Ability Screening Index, HASI	This three-item self-report questionnaire takes 5–10 min to complete for an age range of 13 years to adulthood. Can be administered by professionals with no specialist training. Developed in Australia by Susan Hayes in 2000. Widely used in the UK, Canada, Norway, and The Netherlands.	[27,29,30,31,32,33,34,37,39,42,43,47,68,71]	Sensitivity: 82%, specificity: 70%, validated in prison and forensic settings; quick to administer.
Learning Disability Screening Questionnaire, LDSQ	A seven-item screening tool taking 5–10 min to complete. Can be completed independently, with support, or by someone who knows the person well. Developed in the UK by Karen McKenzie and Donna Paxton in 2006.	[17,33,34,35,43,52,53,54,56,58,79,80]	Sensitivity: 82.3%, specificity: 87.5%; strong internal consistency and inter-rater reliability.
Screener for intelligence and learning disability, SCIL	This 14-item screening tool takes less than 15 min to complete and can be administered by prison staff members without special training. Developed and validated in The Netherlands (2016) by Henk Nijman and colleagues to screen large groups of patients in the CJS.	[34,48,59,60,61,62,63,75,77]	Sensitivity: 76–86%, specificity: 72–83%; validated in criminal justice and psychiatric settings; suitable for use by non-specialists.
Rapid assessment of potential intellectual disability, RAPID	This 15-item self-report questionnaire is quick and simple. Can be administered by staff with little training. Developed in the UK in 2016 by Salma Ali and Scott Galloway.	[25,34]	Initial validation suggests acceptable utility; more research needed on reliability and specificity.
Learning Screening Tool, LST	This seven-item screening tool is quick and efficient to administer and does not require specialist psychological input or staff training. Developed in the UK by Helen Wakeling in 2018.	[65,78]	Early data supports practical utility in correctional settings; limited published psychometric validation.
Knowledge of proverbs, fund of knowledge and similarities. PROFOKS	This three-item brief test of intelligence is quick and easy to administer. Does not require specialised psychological testing expertise. Developed in the USA by Gerard Gallucci and colleagues in 2007.	[38]	Correlates with WAIS; useful in forensic settings; limited published data on broader psychometric performance.
Child and Adolescent Intellectual Disability Screening Questionnaire. CAIDS-Q	This seven-item questionnaire takes 5–10 min to complete. Does not require specialised training or qualifications to use. Developed in the UK by McKenzie, Paxton, Murray, and Milanesi in 2012.	[55]	Cronbach’s alpha: 0.723; statistically significant differences between ID and non-ID groups; good internal validity.

## Data Availability

This scoping review is based on data extracted from publicly available published literature. All sources of data, including databases searched and studies included, are cited within the manuscript. No new or proprietary data were generated during the study. Additional information is available upon request from the corresponding author.
